# Combined Heart and Kidney Transplantation: Initial Clinical Experience

**DOI:** 10.21470/1678-9741-2020-0720

**Published:** 2022

**Authors:** Fernando Antibas Atik, Carolina de Castro Borges, Marcelo Botelho Ulhoa, Renato Bueno Chaves, Vitor Salvatore Barzilai, Rodrigo Santos Biondi, Tiago Martins de Almeida, Isabela Novais Medeiros, Helen Souto Siqueira Cardoso

**Affiliations:** 1 Department of Cardiovascular Surgery, Instituto de Cardiologia do Distrito Federal, Brasília, Federal District, Brazil.; 2 Transplant Unit, Instituto de Cardiologia do Distrito Federal, Brasília, Federal District, Brazil.

**Keywords:** Heart Transplantation, Kidney Transplantation, Heart Failure, Chagas Disease, Cardiomyopathies

## Abstract

**Introduction:**

Combined solid organ transplantation is infrequently performed in Brazil. The objective of this article is to present our initial experience with combined heart and kidney transplantation.

**Methods:**

From January 2007 to December 2019, four patients were submitted to combined heart and kidney transplantation. Their mean age was 55.7±4.4 years, and three (75%) patients were males. All patients had Chagas cardiomyopathy, two were hospitalized and inotrope dependent, and all patients were on preoperative dialysis (median of 12 months prior to transplant).

**Results:**

All patients survived and were in New York Heart Association functional class I at the latest follow-up (mean 34.7±17.5 months). Mean retarded kidney graft function was 22.9±9.7 days. One patient lost the kidney graft two years after the transplant due to Polyomavirus infection.

**Conclusion:**

Our initial experience of combined heart and kidney transplantation was favorable in selected patients with advanced heart failure and end-stage kidney disease. It requires involvement of a dedicated multispecialty team throughout all the diagnostics and treatment steps.

**Table:** 

Abbreviations, Acronyms & Symbols	
ECMO	= Extracorporeal membrane oxygenation
eGFR	= Estimated glomerular filtration rate
LVEDD	= Left ventricular end-diastolic diameter
LVEF	= Left ventricular ejection fraction
LVESD	= Left ventricular end-systolic diameter
NYHA	= New York Heart Association
OR	= Operating room
PASP	= Pulmonary artery systolic pressure
PRA	= Panel-reactive antibodies
PVR	= Pulmonary vascular resistance
RAP	= Right atrial pressure
UNOS	= United Network for Organ Sharing

## INTRODUCTION

Combined solid organ transplantation including the heart is infrequently performed in Brazil. Published medical literature include only anecdotal reports^[1]^, with no publication on the treatment of advanced heart failure and end-stage kidney disease with combined heart and kidney transplantation. Cardiorenal syndrome is frequent in advanced heart failure patients^[2]^. A particular subset of those presents with underlying kidney disease or even prolonged dialysis-dependent acute kidney injury, in which kidney recovery after isolated heart transplantation is less probable. Combined heart and kidney transplantation has been proposed as an attractive alternative of treatment of those patients^[3]^. United Network for Organ Sharing (UNOS) data^[4]^ has shown that the number of patients on the waiting list with this modality of transplant is progressively increasing, as well the number of combined transplants performed.

We aim to present our early experience with combined heart and kidney transplantation in Brazil.

## METHODS

From January 2007 to December 2019, 276 patients were consecutively submitted to orthotopic heart transplantation. Among those, combined heart and kidney transplantation was performed in four patients (1.4%).

Data were retrieved in part from the prospective heart transplantation and kidney transplantation registries and in part from each electronic patient’s medical record. These data were approved by the Institutional Review Board, which also approved their use for research purposes (COI 23681413.5.0000.0026) with patient consent waived.

All patients had documented stage D heart failure with reduced ejection fraction, and they were listed for combined heart-kidney transplantation after fulfilling specific criteria^[5]^ and receiving multidisciplinary team clearance. A consultation with a kidney transplantation team included clinical assessment of perfusion status, relevant hemodynamic parameters (invasive and noninvasive), detection of markers of intrinsic kidney injury, and a thorough investigation of alternative explanations for worsening renal function with renal ultrasonography and biopsy in one patient. A multidisciplinary discussion of risks and benefits aided in the decision to proceed with a combined heart-kidney transplantation. Brazilian Ministry of Health policies on combined transplantation listing and organ allocation determines that these patients must be listed as heart transplantation recipients, using the same criteria as other isolated heart transplantation recipients.

All patients were submitted to complement-dependent cytotoxicity crossmatch before donor acceptance. For that reason, all donors were transferred to and were supported by our hospital in order to ensure appropriate heart function at the time of organ procurement and to minimize cold ischemic times.

Organ procurement was performed in the same hospital as the implantation. All local donors are routinely transferred to our hospital for procurement. Patients were routinely monitored with a pulmonary artery catheter and intraoperative transesophageal echocardiography. First, orthotopic heart transplantation with bicaval anastomosis was performed with normothermic cardiopulmonary bypass and hypothermic antegrade blood cardioplegia for myocardial protection. Just after the end of heart transplantation, with the chest closed, kidney transplantation was commenced. Induction therapy with thymoglobulin was used in all patients, and the maintenance regimen was tacrolimus, azathioprine, and prednisone.

Postoperative management followed institutional protocols, which include early extubation, hemodynamic support aiming cardiac index > 2.5 L/min/m2, central venous pressure < 10 mmHg, and pulmonary wedge pressure < 10 mmHg. Diuresis was > 50 ml/hour. Cardiac function is verified with bedside transthoracic echocardiography in case of clinical suspicion or altered parameters with invasive monitoring. Follow-up was obtained through medical consultations and appropriate tests. Surveillance endomyocardial biopsies were performed weekly in the first month, then monthly in the next five months, and every six months thereafter. Diagnosis of rejection follow the International Society for Heart and Lung Transplantation classification. We have completed follow-up of all patients.

## RESULTS

Patients’ mean age was 55.7±4.4 years and three (75%) of them were males. Data is summarized in Table 1. All patients were in New York Heart Association (NYHA) functional class IV and Chagas cardiomyopathy was the primary cause of cardiac disease. Echocardiography revealed severely dilated left ventricles (mean end-diastolic diameter of 70±5.7 millimeters and mean end-systolic diameter of 58±7 millimeters) and poor function (mean ejection fraction of 22.4%±3.4%). Right heart catheterization showed a mean cardiac index of 1.6±0.5 L/min/m^2^, mean systolic pulmonary artery pressure of 51.5±7.9 mmHg, mean right atrial pressure of 19±6.7 mmHg, and mean pulmonary vascular resistance of 4.5±2.4 Wood units. Patients remained on the waiting list a mean of 138±97 days, and two of them were on priority status at the time of transplantation, being inotrope dependent.

**Table 1:** Distribution of patient characteristics.

	Patient 1	Patient 2	Patient 3	Patient 4
Gender	Male	Male	Female	Male
Age (years)	56	62	54	51
Cardiac etiology	Chagas	Chagas	Chagas	Chagas
Kidney etiology	Cardiorenal	Cardiorenal	Undetermined	Pesticide-induced
NYHA Class	IV	IV	IV	IV
Inotrope dependence	Yes	Yes	No	No
Time on dialysis (months)	2	2	22	214
Time on waiting list (days)	94	75	290	134
PRA (%)	0	0	45	22
Preoperative creatinine (mg/dl)	2.2	2.3	7.4	6.7
eGFR (mL/min/m2)	32	36.2	6.5	12.9
LVEF (%)	22.3	27.2	20	20
LVEDD (mm)	76	68	63	73
LVESD (mm)	61	55	50	66
Cardiac index (L/min/m2)	1.08	1.51	1.62	2.32
PASP (mmHg)	40	55	53	58
RAP (mmHg)	18	23	10	25
PVR (Wood units)	2.38	3.66	8 [3.63*]	3.88 [1.36*]
Recipient blood type	O	O	O	A
Donor blood type	O	O	O	A
Donor sex	Male	Male	Female	Male
Donor age (years)	18	20	20	21

eGFR=estimated glomerular filtration rate; LVEDD=left ventricular end-diastolic diameter; LVEF=left ventricular ejection fraction; LVESD=left ventricular end-systolic diameter; NYHA=New York Heart Association; PASP=pulmonary artery systolic pressure; PRA=panel-reactive antibodies; PVR=pulmonary vascular resistance; RAP=right atrial pressure

* values between brackets with infusion of sodium nitroprusside

All patients were on dialysis (median of 12 months) prior to transplant. Mean serum creatinine was 4.7±2.8 mg/dl and mean glomerular filtration rate was 20.4±14.4 mL/min/m2. Etiology of kidney disease was cardiorenal syndrome in two patients, organophosphate pesticide-induced nephrotoxicity in one patient, and undetermined in the last.

Two patients had had panel-reactive antibodies prior to transplant. Mean donor age was 19.7±1.2 years and three (75%) were males. The causes of death were head trauma in three (75%) and hemorrhagic cerebrovascular accident in the last. Donors did not have any important comorbidities, and only one had active infection (nosocomial pneumonia). Mean cold ischemic times were 68.7±15.6 minutes. One patient developed severe primary graft dysfunction in the operating room because he failed to wean off cardiopulmonary bypass and required extracorporeal membrane oxygenation (ECMO). Kidney transplantation was performed with the patient on ECMO.

Postoperatively, median duration of hospitalization was 52 days. Mean serum creatinine at hospital discharge was 1.4±0.5 mg/dl, mean retarded kidney graft function was 22.9±9.7 days, with an average of 23.7±2.6 sessions of post-transplant dialysis. One patient had kidney 2R acute cellular rejection two months after transplant, and active cytomegalovirus infection occurred in two patients. Mean follow-up was 34.7±17.5 months (variation 22 - 48 months). There was no death. One patient lost the kidney graft two years after the transplant due to Polyomavirus infection. All patients were in NYHA functional class I at the latest follow-up.

## COMMENTS

The present data shows a single-center initial experience on combined heart-kidney transplantation in Brazil, which is the first published experience to date. Our data relates to a population of Chagas cardiomyopathy patients with stage D heart failure refractory to medical treatment. Both noninvasive and invasive markers of disease severity were consistent with the need for heart transplantation. Chagas cardiomyopathy is associated with frequent biventricular dysfunction^[6]^ and arrhythmia-related sudden death, being an independent predictor of transplant waitlist mortality^[7]^.

Concomitant important kidney disease was determined in all patients because all were on dialysis (median of 12 months prior to transplant), among other findings. However, there were two different clinical profiles in our experience. Chronic primary kidney disease with long-term outpatient dialysis was present in patients 3 and 4. They were listed and waited at home with no signs of end-organ dysfunction. On the other hand, patients 1 and 2 presented with cardiogenic shock and end-organ dysfunction, with cardiorenal syndrome. They were initially stabilized with intravenous inotropes and developed worsening kidney function, with the need for hemodialysis. The decision to proceed with the combined transplant was driven by a combination of factors (need for dialysis ~ 2 months, low glomerular filtration rate, and small kidneys on abdominal ultrasound and biopsy in one patient). The decision was shared by a multidisciplinary panel of specialists. One may argue that bridging these patients with durable mechanical devices would potentially reverse end-organ dysfunction, opening the possibility of an isolated heart transplant in the future. Although this strategy is standard of care in many countries, it seems complicated in Brazil due to the unavailability of devices in the Brazilian public health system. Moreover, the type of mechanical support would be potentially a biventricular assist device or total artificial heart, giving the heart failure etiology.

The most important issue to be discussed is patient selection; central to a discussion of combined organ transplantation is the question of whether native renal function will improve following heart transplantation alone. Kidney transplantation can be requested at time of heart transplantation or years after; however, no consensus exists regarding indications and timing for kidney transplant. A combined heart and kidney transplantation approach for treating coexisting end-organ failure at the time of heart transplantation has shown increased survival for both dialysis-dependent patients and patients with reduced preoperative glomerular filtration rate^[8]^.

However, there are no uniformly agreed upon criteria for selecting a combined organ transplant strategy over sequential or isolated organ transplant. A threshold of estimated glomerular filtration rate (eGFR) has been searched by several investigators as a guide that potentially justified combined transplantation. Previous studies recommend combined heart and kidney transplantation for patients with renal function in the interval from 33 mL/min^[9]^ to 37 mL/min^[10]^, but there is still no consensus about the threshold below which the combined procedure would be recommended. The International Society for Heart and Lung Transplantation consensus guidelines from 2016 do list an eGFR < 30 mL/min/m2 as a relative contraindication to heart transplantation (Class IIa, level of evidence: C), which serves as recommendation for combined heart-kidney transplantation^[11]^.

The most recent publication from the UNOS database^[12]^ shows that patients with a preoperative eGFR < 30 mL/min or in dialysis achieved a longer survival with combined heart-kidney transplantation compared with sequential kidney transplantation after heart transplantation. Patients who required the latter strategy are usually those who after heart transplantation develop kidney failure due to calcineurin inhibitor nephrotoxicity. The median time between heart and kidney transplant was six years. All of our patients were on preoperative dialysis and had a mean eGFR of 20.4±14.4 mL/min, which supports the indication for combined transplantation.

Cardiorenal syndrome was the most frequent cause of kidney failure in our patients. Because of combined transplantation is infrequently performed in our country, it is important to recognize that the eligibility of patients that would potentially benefit from this strategy is underappreciated in Brazil. Concurrent allocation of dual organs in these recipients appears appropriate and this has important implications for organ allocation, particularly because it utilizes two organs in one recipient. In Brazil, patients listed for combined heart and kidney transplantation remain in the heart transplantation listing with the same criteria of allocation and prioritization organized and regulated by the Ministry of Health. There are ethical issues related to combined transplantation due to scarcity of organs for every recipient. For that reason, strict and precise indications for combined transplantation are of paramount importance, particularly because of high mortality rates observed in advanced heart failure patients on the waiting list for isolated heart transplantation. Similar debate prevails in the indication of single lung transplantation in certain pulmonary diseases that enhances the number of treated patients, as opposed to double lung transplantation improves survival on less treated patients^[13]^.

It is certainly a more complex group of patients in terms of disease severity, and it is more complex to perform. It requires two surgical teams that do operate in sequence and it requires though a coordinated team with prolonged operating room time. In our center, we decided to limit to local donors only in order to minimize cold ischemic times, to test for real crossmatch, and to use induction therapy with thymoglobulin as a routine. On the other hand, there is experimental evidence to suggest that the greater mass of transplanted tissue may reduce rejection rates and promote graft tolerance^[14]^.

## CONCLUSION

**Fig. 1:**
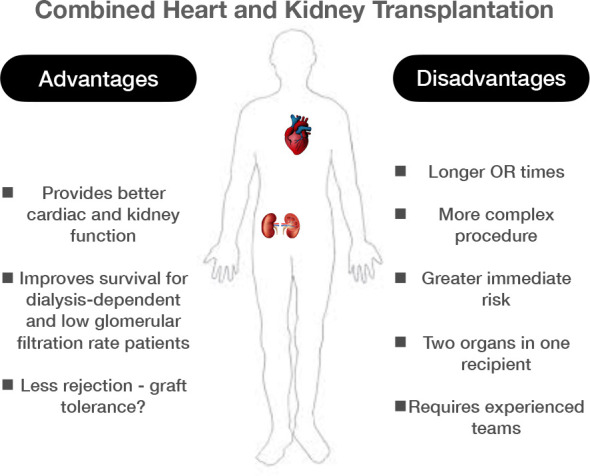
Combined heart and kidney transplantation: advantages and disadvantages. OR=operating room.

In conclusion, our initial experience of combined heart and kidney transplantation was favorable in selected patients with advanced heart failure and end-stage kidney disease (Figure 1). It requires involvement of a dedicated multispecialty team throughout all the diagnostics and treatment steps.

**Table:** 

Authors’Roles & Responsibilities	
FAA	Substantial contributions to the conception or design of the work; or the acquisition, analysis, or interpretation of data for the work; drafting the work or revising it critically for important intellectual content; agreement to be accountable for all aspects of the work in ensuring that questions related to the accuracy or integrity of any part of the work are appropriately investigated and resolved; final approval of the version to be published
CCB	Substantial contributions to the conception or design of the work; or the acquisition, analysis, or interpretation of data for the work; drafting the work or revising it critically for important intellectual content; agreement to be accountable for all aspects of the work in ensuring that questions related to the accuracy or integrity of any part of the work are appropriately investigated and resolved; final approval of the version to be published
MBU	Drafting the work or revising it critically for important intellectual content; agreement to be accountable for all aspects of the work in ensuring that questions related to the accuracy or integrity of any part of the work are appropriately investigated and resolved; final approval of the version to be published
RBC	Drafting the work or revising it critically for important intellectual content; agreement to be accountable for all aspects of the work in ensuring that questions related to the accuracy or integrity of any part of the work are appropriately investigated and resolved; final approval of the version to be published
VSB	Drafting the work or revising it critically for important intellectual content; agreement to be accountable for all aspects of the work in ensuring that questions related to the accuracy or integrity of any part of the work are appropriately investigated and resolved; final approval of the version to be published
RSB	Substantial contributions to the conception or design of the work; or the acquisition, analysis, or interpretation of data for the work; drafting the work or revising it critically for important intellectual content; agreement to be accountable for all aspects of the work in ensuring that questions related to the accuracy or integrity of any part of the work are appropriately investigated and resolved; final approval of the version to be published
TMA	Substantial contributions to the conception or design of the work; or the acquisition, analysis, or interpretation of data for the work; drafting the work or revising it critically for important intellectual content; agreement to be accountable for all aspects of the work in ensuring that questions related to the accuracy or integrity of any part of the work are appropriately investigated and resolved; final approval of the version to be published
INM	Drafting the work or revising it critically for important intellectual content; agreement to be accountable for all aspects of the work in ensuring that questions related to the accuracy or integrity of any part of the work are appropriately investigated and resolved; final approval of the version to be published
HSSC	Substantial contributions to the conception or design of the work; or the acquisition, analysis, or interpretation of data for the work; drafting the work or revising it critically for important intellectual content; agreement to be accountable for all aspects of the work in ensuring that questions related to the accuracy or integrity of any part of the work are appropriately investigated and resolved; final approval of the version to be published
